# Evaluation of Acrylamide/α-Lipoic Acid Statistical Copolymers as Degradable Water-Soluble Kinetic Gas Hydrate Inhibitors

**DOI:** 10.3390/polym17233125

**Published:** 2025-11-25

**Authors:** Chong Yang Du, Milan Marić, Phillip Servio

**Affiliations:** Department of Chemical Engineering, McGill University, Montreal, QC H3A 0C5, Canada; chong.du@mail.mcgill.ca (C.Y.D.); milan.maric@mcgill.ca (M.M.)

**Keywords:** reversible-deactivation radical polymerization, ring-opening polymerization, water-soluble polymer, degradable polymer, alpha-lipoic acid, hydrate inhibitor

## Abstract

Readily degradable low-dose hydrate inhibitors are of great significance for flow assurance in the petroleum industry. Recently, α-lipoic acid (LA) was shown to undergo ring-opening reaction via reversible addition–fragmentation chain-transfer copolymerization with acrylamides to introduce labile disulfide bonds into the stable vinyl polymer backbone. Here, LA was copolymerized with acryloyl morpholine (AM) to evaluate their performance as kinetic hydrate inhibitors. Degradability was confirmed for the copolymers with 20 mol.% LA (AM/LA20, *M*_n_ = 19 → 9 kDa) after disulfide reduction. Thermogravimetric analysis also indicated faster thermal degradation of AM/LA due to the incorporation of weaker S-S and S-C linkages. Increasing LA content reduced hydrophilicity, and the copolymers were treated with NaOH to ensure water solubility. However, at 700 ppm, poly(AM) homopolymer reduced methane consumption during hydrate growth to 54% with respect to the uninhibited system, while gas consumption for the carboxylate AM/LA20 reached 78%. An advantageous feature of LA is its carboxylic acid, allowing desired functionalities to be grafted onto the degradable copolymer. Isopropyl amine (IPAm) was coupled with LA to form an amide known to be effective during hydrate inhibition (LA(IPAm)). The copolymer AM/LA(IPAm)20 demonstrated better water solubility compared to the original AM/LA20. Furthermore, the desirable IPAm functionality allowed the hydrate inhibition to be re-established at 54%, nearly recovering the performance of the poly(AM) homopolymer. This article assesses the application of LA and LA derivatives as building blocks for degradable amide-based kinetic hydrate inhibitors by validating their degradability with material characterizations and their inhibition performance during structure I hydrate growth.

## 1. Introduction

In offshore oil and gas facilities, gas hydrate agglomeration is a pressing concern for flow assurance [1]. The extreme pressures, low temperatures, and water content in petroleum pipelines are favorable to formation of gas hydrates, which are clathrate compounds composed of small gas molecules trapped within water lattices [2]. The growth and accumulation of these crystalline solid particles can lead to pipeline blockage, production loss, and safety hazards [3]. Typical strategies for flow assurance include injections of copious quantities of anti-freeze compounds such as alcohols and glycols into the system to disrupt the water molecules’ hydrogen-bonding network [4]. This method comes with high operating costs and environmental concerns, and thus the development of low-dose hydrate inhibitors (LDHIs) has been very active since the 1970s [5].

Thermodynamic inhibitors such as methanol would need to be loaded at a level of at least 20 wt.% to be effective, whereas LDHIs are typically required at less than 1 wt.% [5]. Amongst LDHIs, kinetic hydrate inhibitors (KHIs), a class consisting of water-soluble polymers able to both delay hydrate nucleation and to reduce hydrate growth rate, are of acute interest. Although applied in low concentrations in the system, discharge of KHIs in the aquatic environment and their impacts on marine life cannot be overlooked [6]. Since they are not present in the form of solid particles, synthetic water-soluble polymers such as poly(vinylpyrrolidone) (PVP) and poly(acrylamides) are classified separately from microplastics [7]. Compared to other common plastics such as poly(styrene) (PS), poly(vinyl chloride) (PVC), and poly(ethylene) (PE), recognition of the necessity of degradable alternatives for water-soluble polymers in the aquatic environment has only occurred recently [8].

Rajput et al. tested the methane hydrate inhibition ability of poly(vinyl alcohol) (PVA), one of the few vinyl polymers which can offer biodegradability in the presence of specific microorganisms [9,10]. Compared to PVP, which is one of the most commercially available and researched KHIs, PVA exhibited a much weaker inhibition effect, potentially due to their drastically smaller side groups. Wan and Liang have achieved partially degradable KHIs by first synthesizing hydroxyl-terminated poly(vinylcaprolactam) (PVCap), followed by ring-opening polymerization of ε-caprolactone [11]. Their amphiphilic block copolymers have shown improved hydrate inhibition compared to PVCap homopolymer. Unlike classical step-wise chemistries, introducing degradable ester linkages into vinyl polymer backbones is rarer. One method to introduce such degradation sites is by ring-opening polymerization of cyclic ketene acetals (CKAs) [12]. Free-radical copolymerization of 5,6-benzo-2-methylene-1,3-dioxepane (BMDO) with *N*-isopropylacrylamide (NIPAm) resulted in P(NIPAm-co-ester)s that undergo hydrolytic degradation [13]. These P(NIPAm-co-ester)s have not been tested as hydrate inhibitors, although PNIPAm homopolymers demonstrated comparable performance to the commercially available KHI of the 1:1 statistical copolymer of *N*-vinylpyrrolidone/*N*-vinylcaprolactam (NVP/NVCap, Luvicap 55W) [14,15]. Other than degradable synthetic polymers, naturally occurring molecules such as polysaccharides, namely chitosan and its derivatives, and amino acids have displayed abilities to reduce gas uptake during hydrate growth [16,17,18,19,20,21]. Related to amino acids, polyaspartamides, derived from polysuccinimide, showed good biodegradability and behaved as good inhibitors for structure II hydrates, although slightly weaker than Luvicap 55W [22].

**Figure 1:**
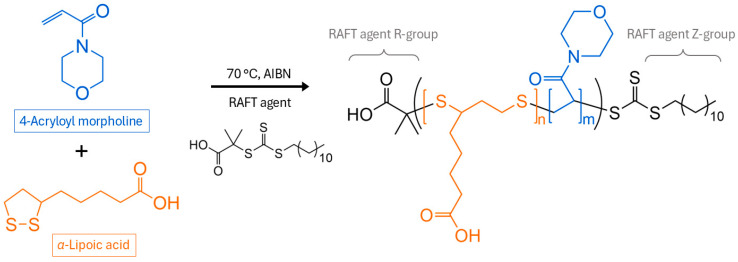
Reaction scheme of ring-opening reaction of α-lipoic acid with acryloyl morpholine via RAFT polymerization, as well as the RAFT agent structure.

In this work, a comonomer that can impart degradability into vinyl-based polymers—α-lipoic acid (LA), a common dietary supplement—was investigated for their application as polymeric hydrate inhibitors. In a recent article, Hawker and co-workers presented LA’s ability to undergo radical ring-opening polymerization (ROP) using reversible-deactivation radical polymerization (RDRP) in the presence of acrylates and acrylamides [23]. Copolymerization with LA incorporates disulfide bonds into the carbon backbone, and these are degradable under mild conditions. Various acrylamides possess the characteristics of hydrate inhibitors [24,25]. They are a relatively newer class of KHIs, and acrylamides have also acted as great synergists to vinyl amides to further delay hydrate induction and to reduce crystal growth [26,27]. In this work, acryloyl morpholine (AM) was selected to be copolymerized with LA to investigate the feasibility of introducing degradability into vinyl-based KHIs. The polymerization reaction scheme is illustrated in Figure 1.

## 2. Materials and Methods

### 2.1. Materials

Hereby presented is the list of chemicals used in this project with their abbreviations, purities, and suppliers: 4-Acryloyl morpholine (AM, 97%, Sigma-Aldrich, St. Louis, MO, USA), DL-α-lipoic acid (LA, >99.0%, TCI America, Portland, OR, USA), 2-(Dodecylthiocarbonothioylthio)-2-methylpropionic acid (RAFT agent, high-performance liquid chromatography (HPLC) grade, 98%, Sigma-Aldrich), Azobisisobutylnitrile (AIBN, recrystallized), Acetonitrile (MeCN, Certified ACS, Fisher Chemical, Waltham, MA, USA), *N*,*N*-Dimethylformamide (DMF, HPLC grade, ≥99.9%, Sigma-Aldrich), Diethyl ether (≥99.9%, Sigma-Aldrich), Tetrahydrofuran (THF, HPLC grade, ≥99.9%, Sigma-Aldrich), Heptane (HPLC grade, Fisher Chemical), Tris(2-carboxyethyl)phosphine hydrochloride (TCEP, Sigma-Aldrich), Chloroform (anhydrous, ≥99%, stabilized with amylenes, Sigma-Aldrich), Sodium hydroxide (NaOH, pellets, Certified ACS, Fisher Chemical), Reverse osmosis water (RO water, with resistivity of 1.2 MΩ), Nitrogen (Smart Top, ALPHAGAZ^TM^, Air Liquide Canada, Montreal, QC, Canada), Methane (ultra high purity grade, 99.97%, Linde Canada, Inc., Montreal, QC, Canada).

### 2.2. Polymer Synthesis and Characterization

The AM monomer was filtered through a column comprising activated basic alumina (Al_2_O_3_, 1 g per 50 mL of monomer) and calcium hydride (CaH_2_, 5 wt.% of the basic alumina) to remove any inhibitor and protic impurities. The purified monomer was subsequently stored under nitrogen. The poly(acryloyl morpholine) (PAM) homopolymer and the AM/LA statistical copolymers were synthesized using reversible addition–fragmentation chain-transfer (RAFT) polymerization to effectively control the molecular weight distribution and observe degradation more clearly. The RAFT agent selected was 2-(dodecylthiocarbonothioylthio)-2-methylpropionic acid, which was used for the controlled radical polymerization of LA with various acrylates by Albanese et al. [23]. The target molecular weight was 40 kg/mol at 100% theoretical conversion. The radical initiator AIBN was added at 10 mol.% with respect to the amount of RAFT agent, and the monomer mixture was diluted using 50 vol.% of MeCN or DMF as solvent. The reaction was conducted at 70 °C in a 10 mL three-neck round-bottom flask under nitrogen purge until the reaction mixture reached very high viscosity. Polymers were first precipitated using diethyl ether, then redissolved in THF and reprecipitated using heptane to remove the unreacted monomers and solvent. As an example, AM/LA10 was synthesized by adding 2.37 g of AM, 0.384 g of LA, 25.3 mg of RAFT agent, 1.1 mg of AIBN, and 2.7 g of MeCN into the reactor. The recovered copolymer weighed 1.35 g (yield = 49%), partly due to the relatively large number of samples taken during the reaction for characterization, although it could also be attributed to the partial solubility of LA in a wide variety of solvents. Hawker and coworkers chose to purify using dialysis in acetone (1 L × 2) to increase the yield.

Aliquots of 0.1 mL were taken from the reactor at fixed intervals during the polymerization reaction. The monomer conversions in the Results section, as well as the final LA composition in the copolymers, were obtained from ^1^H NMR spectra in chloroform-d, using Bruker Avance III HD 500 MHz NMR Spectrometer. ^1^H NMR (*δ*, in CDCl_3_, 500 MHz): 1.0–1.4 and 1.4–2.0 ppm (2H, -C*H*_2_-CH-, AM backbone); 1.3–1.7 ppm (6H, -C*H*_2_-C*H*_2_-C*H*_2_-CH_2_-COOH, LA side chain); 1.8–2.0 ppm (2H, -S-CH_2_-C*H*_2_-CH-S-, LA backbone); 2.0–2.3 ppm (2H, -CH_2_-CH_2_-CH_2_-C*H*_2_-COOH, distinct LA peak in copolymer); 2.3–2.8 ppm (1H, -CH_2_-C*H*-, AM backbone); 2.7–3.0 ppm (3H, -S-C*H*_2_-CH_2_-C*H*-S-, LA backbone); 3.1–4.0 ppm (8H, morpholine ring, distinct AM peak in copolymer).

Molecular weights and dispersities (*Đ*) were determined using gel permeation chromatography (GPC). The eluent consisted of HPLC-grade DMF at 50 °C containing 10 mM LiBr. Test solutions with a concentration of 3 mg/mL were filtered through a 0.22 µm filter, and aliquots of 30 μL were injected and eluted at a flow rate of 0.4 mL/min. The column used was a Shodex SHSB-806MHQ (Shodex, Tokyo, Japan), 300 mm × 8 mm, with 13 μm particle size, and a Shimadzu RID-20A refractive index detector (Shimadzu, Kyoto, Japan) was applied. Calculations of molar mass and *Đ* were performed against a calibration curve constructed with PMMA standards (Agilent (Santa Clara, CA, USA), 1780 to 265,300 g/mol). Prior to using this setup, a different GPC instrument (Water Breeze, Milford, MA, USA) equipped with ResiPore columns (250 mm × 4.6 mm, 3 μm particle size) was tested for molecular weight measurements. The latter, however, was unable to provide sufficient resolution to separate the peaks of samples containing LA.

Thermal degradation curves were measured using a TA Instruments TGA, model Discovery 5500, at a heating ramp of 15 °C/min from 25 °C to 600 °C under nitrogen. Thermal transitions via heat/cool/heat experiments were performed using a TA Instruments DSC, model Discovery 2500, ramping at 10 °C/min under nitrogen from 0 °C to a varying maximum temperature for each polymer, which did not exceed the 5% weight loss decomposition temperature measured previously using TGA. Glass transition temperatures (*T*_g_) were calculated using the half-height analysis on the glass transition region of the second heat cycle.

### 2.3. Degradability and Water Solubility Treatments

To test the degradability of the copolymers containing disulfide bonds, the AM/LA copolymers were first dissolved in a mixture of THF and water (50:50 volume ratio) in a 10 mL three-neck reactor. TCEP was added to the solution at 1 mol equivalent relative to the amount of LA repeat units. The mixture was stirred overnight at 60 °C under nitrogen purge. Resulting polymers were recovered from the aqueous solution by washing with 10 mL of chloroform. The solvent was evaporated first in the fume hood, and then in a vacuum oven at 40 °C and −30 in Hg. Purified degraded polymers were inspected using Fourier transform infrared spectroscopy (FTIR) to ensure no trace of TCEP remained. A Thermo Scientific Nicolet IS50 FTIR spectrophotometer was used to obtain the spectra via a diamond attenuated total reflectance (ATR) using 32 scans.

To enhance water solubility, the AM/LA copolymers were treated with 1 M NaOH solution until all the carboxylic acids from the LA repeat units were converted into their sodium carboxylate forms. The completion of the deprotonation reaction was indicated by the complete dissolution of AM/LA copolymers in reverse osmosis-purified water (RO water) under stirring, as well as attainment of basic pH of the aqueous solution. The excess NaOH was removed via dialysis in 4 batches of 2 L RO water, using SnakeSkin dialysis tubing from Thermo Fisher Scientific (Waltham, MA, USA), 3.5K MWCO, 35 mm I.D., until a neutral pH was observed. Water was removed via lyophilization at −80 °C and 0.2 Torr, and the deprotonated copolymers were analyzed using FTIR to ensure the entire COOH stretch had shifted to become a COO- peak. Dynamic light scattering (DLS) was used to determine the polymer particle sizes in RO water. Solutions at concentration of 1 wt.% were loaded in DTS0012 disposable cuvettes, and measurements were performed using Malvern Panalytical Zetasizer Nano ZS equipped with a 633 nm laser, assuming the material properties of a polystyrene latex (RI: 1.590, Absorption: 0.010).

### 2.4. Hydrate Growth Experiments

**Figure 2:**
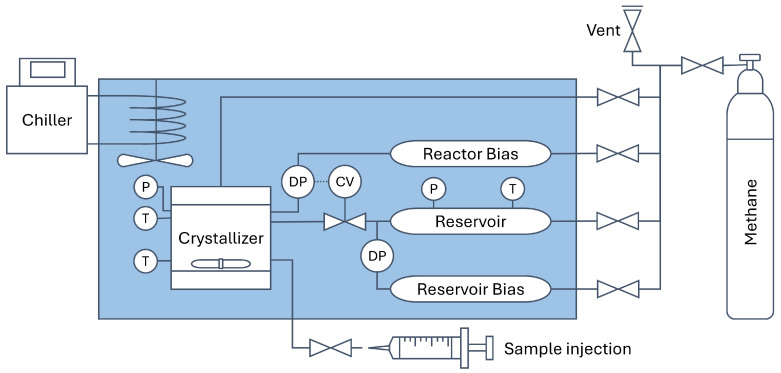
Simplified schematic of the methane hydrate kinetic experiment setup. Acronyms: T is for temperature probe (thermocouple), P is for pressure transducer, DP is for differential pressure transducer, and CV is for control valve.

The setup for methane hydrate growth experiments has been described in detail in previous reports investigating gas hydrate growth kinetics [9,28,29,30,31]. The schematic of the setup is illustrated in Figure 2. The experimental conditions were 4646 kPa and 2 °C, which corresponds to a 1500 kPa pressure driving force for methane hydrates. Each test solution, including RO water control, was tested 5 times. The test conditions were selected to be able to make direct comparisons with previous reports using the same experimental setup [9,29,32].

Test solutions (300 mL) were prepared by dissolving 700 ppm by weight of polymer in RO water, which was stirred for 24 h. They were injected into a 316 stainless steel crystallizer (pressure rating of 12 MPa) and brought to the temperature setpoint. The system was fully submerged in a 50/50 volume mixture of ethylene glycol and water, which is contained within an insulated tank. The test solution temperature in the reactor was kept to within ±0.1 °C of the setpoint.

Purging was performed by pressurizing the system with 2000 kPa of methane and depressurizing to 150 kPa for five cycles. The reactor was then pressurized to an operating pressure of 4646 kPa. The reservoir, the reactor bias, and the reservoir bias cylinders were pressurized to 5646 kPa. When temperatures and pressures had equilibrated, data recording began in LabVIEW, and the magnetic stirrer in the crystallizer was turned on to induce gas dissolution. The growth experiments were isobaric. As methane was being consumed in the reactor, cold methane gas was supplied from the 1000 cm^3^ reservoir by a Baumann 51,000 Series control valve (CV) to keep the differential pressure (DP) constant between the reactor and reactor bias (±2 kPa). The amount of methane consumed was recorded by measuring the DP between the reservoir and reservoir bias.

**Figure 3:**
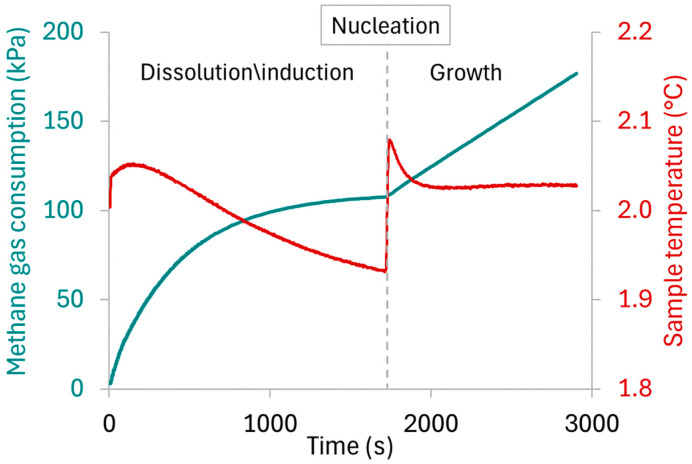
Representative graph of pressure and temperature profiles measured during a methane hydrate growth experiment, marking the dissolution/saturation, nucleation, and hydrate growth events.

Hydrate formation is exothermic and can be identified by an increase in test solution temperature and a change in slope in the gas consumption curve, as indicated in Figure 3. As nucleation is stochastic, methane consumption rates were measured as the slope during the growth phase. From the reservoir pressure and temperature measurements, as well as its volume, the number of moles of methane consumed were calculated using the Trebble–Bishnoi equation of state. After 30 min of hydrate growth, the CV was turned off, and the reactor was slowly depressurized while letting the stir bar break down the hydrate crystals. The reactor was rinsed 5 times by injecting 360 mL of RO water and stirring for one minute with each rinse.

## 3. Results and Discussion

**Figure 4:**
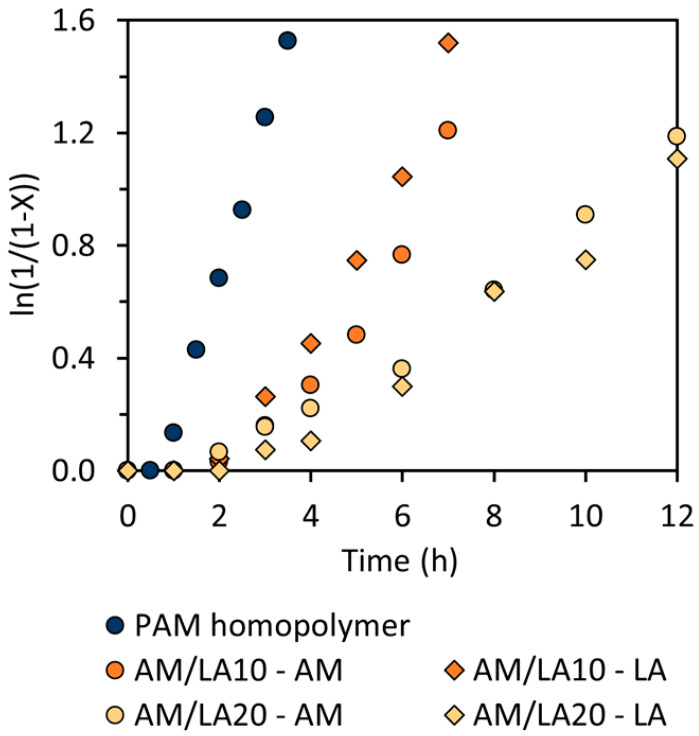
Linearized semi-logarithmic first-order kinetic plot of conversion *X* versus time for PAM homopolymer and AM/LA copolymers at 70 °C.

As presented in Table 1, the copolymerization of AM and LA was performed in a controlled manner (*Đ* < 1.5) [33]. As shown in Figure 4, the polymerization rate became increasingly slower for copolymerization compared to the AM homopolymerization as the feed composition of LA increased. At 10 mol.% AIBN to RAFT agent ratio, the PAM homopolymer exhibited 30 min of retardation, which rose to 1 h for AM/LA copolymers. For RAFT polymerization, the phenomenon of retardation is not uncommon, being most prevalent in reactions using dithiobenzoates for styrene and methyl acrylates [34,35,36,37]. Trithiocarbonates, such as those illustrated in Figure 1, are typically more stable and less prone to side reactions such as hydrolysis. Although, due to the high propagation rate constants of acrylates, acrylamides, and vinyl amides, some reports suggest that primary or secondary R-groups might be more suitable than the tertiary R-group from Figure 1 [38]. Investigations into the compatibility of these other RAFT agents with the LA monomer can constitute a separate study.

**Figure 5:**
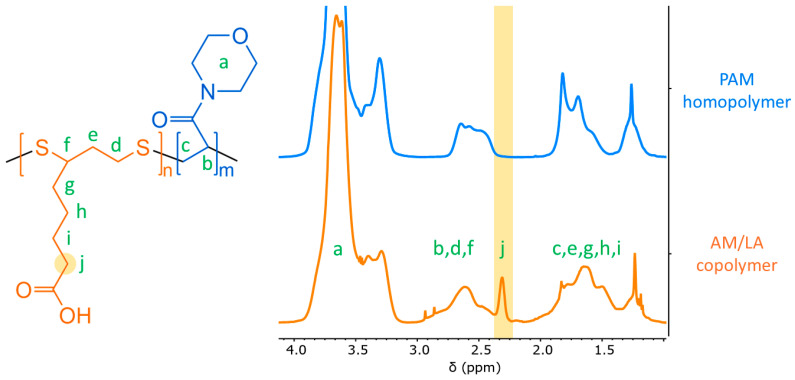
^1^H NMR spectra of PAM homopolymer and AM/LA10 copolymer in CDCl_3_ (500 MHz). The lowercase letters attribute each proton to their corresponding peak within the spectra. The characteristic peak for the LA repeat-units within the copolymer, used to determine the final copolymer composition *F*_LA_, has been highlighted.

Due to the broad morpholine signal at 3.1–4.0 ppm (8H) in the ^1^H NMR spectrum, methylation of the carboxylic acid group in the LA repeat unit is an ineffective method for composition characterization (3.6 ppm) [23]. Nevertheless, a distinct LA peak can still be identified at 2.0–2.3 ppm (2H), as indicated in Figure 5. The LA feed composition (*f*_0,LA_) and final composition in the copolymer (*F*_LA_) compiled in Table 1 match relatively well. The conversion curves of AM and LA in Figure 4 also suggest a relatively random repeat unit pattern, reflected by the comparable kinetics of the two monomers during copolymerization, which is sought after to improve the likelihood of evenly distributed disulfide bonds across the chain, as opposed to a more gradient-like arrangement along the backbone. As a result, a gradient microstructure is improbable, as LA alone seems unlikely to be controlled by the chain-transfer agent (CTA), and it requires an acrylate or acrylamide comonomer to proceed to ring-opening polymerization [23]. Indeed, this study shows that LA only begins to polymerize in conjunction with AM despite the latter’s retardation behavior.

**Table 1:** Reaction conditions and characterization results for PAM and AM/LA copolymers.

Polymer ID ^1^	Solvent at 50 wt.%	Time (h)	*f*_0, LA_^2^	*F*_LA_^3^	*X *^4^ (%)	*M*_n_^5^ (kg/mol)	*Đ * ^5^
PAM	MeCN	3.5	-	-	78	20.3	1.26
AM/LA10	MeCN	7.0	10	12	71	19.4	1.37
AM/LA20	DMF	12.0	20	22	69	18.6	1.47

^1^. PAM: poly(acryloyl morpholine) homopolymer; AM/LAxx: xx mol.% LA in the copolymer; ^2^. Initial molar fraction of LA in the feed; ^3^. Final molar fraction of LA in the copolymer, determined using proton NMR; ^4^. Final conversion, determined using proton NMR: *X* = *X*_AM_*f*_0,AM_ + *X*_LA_*f*_0,LA_; ^5^. Number average molecular weight and dispersity, determined using DMF GPC with 10 mM LiBr at 50 °C, against a PMMA calibration curve.

Comparable molecular weights of the polymer products were achieved despite their notably different reaction kinetics, providing a fair comparison during hydrate growth experiments. The effect of molecular weight on hydrate growth was reported by Posteraro et al., where methane consumption in an inhibited system showed no significant difference with PVP of molecular weights of 10, 40, and 360 kg/mol [29]. Likewise, in a study comparing the inhibition performance of PVP, poly(vinyl piperidone) (PVPip), and PVCap, the difference between polymers far exceeded the small effects of molecular weights for a particular polymer [39,40].

**Figure 6:**
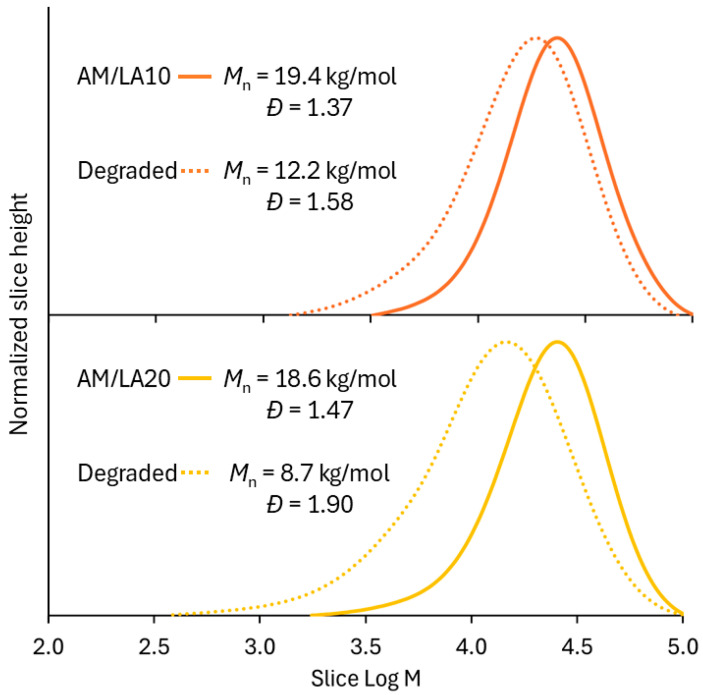
GPC slice data of AM/LA copolymers before and after treatment with TCEP, using DMF eluent with 10 mM LiBr at 50 °C, calculated against a PMMA calibration curve.

**Figure 7:**
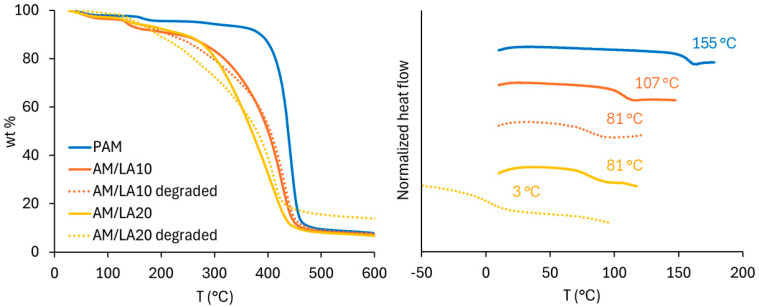
Thermal analysis of PAM homopolymer and AM/LA copolymers before and after TCEP reduction: TGA (**left**) and DSC (**right**).

Figure 6 presents the molecular weight distributions of the AM/LA copolymers after TCEP reduction. Degradation of LA copolymers into smaller segments was successful, as shown by shifts in the GPC chromatograms. The *M*_n_ of degraded AM/LA copolymers at 10% and 20% LA feed (12 kg/mol and 9 kg/mol, respectively) are comparable to those shown in the first report by Albanese et al. in 2023 [23]. Data obtained from the thermal analysis are shown in Figure 7. TGA curves further confirm the copolymers’ enhanced degradability, as higher content of LA leads to much faster weight reduction during the temperature ramp. In this work, a shift in GPC traces was observed following a S-S cleavage using TCEP, which has the advantages of requiring a low-toxicity aqueous environment and mild reduction temperatures [41]. A very recent article by Hawker’s group pointed out that the thermal reduction in S-C, a much more abundant linkage than S-S within the polymer backbone, leads to significantly lower *M*_n_ than that obtained from a disulfide reduction at mild conditions when reacted in DMF at elevated temperatures [42]. The same degree of degradation obtained at 20 mol.% LA using TCEP disulfide reduction can be achieved by degrading S-C bonds at 100–140 °C for copolymers with only 2.5 mol.% LA content.

Multiple research articles have exploited the dynamic activation of disulfide bonds in LA, poly(LA), and their derivatives under irradiation at room temperature [43,44,45,46,47]. As an antioxidant, LA also easily chelates metals and quenches radicals [48]. Because the monomer is sourced from plants and animals, poly(LA) can also be classified as biodegradable, as it can be cleaved by in vivo reducing agents [49]. Coupled with signs of decomposition from various characterization methods, this compound is a very promising building block for degradable materials. However, it is important to note that the degradation has been performed in a laboratory setting. To truly confirm the actual environmental degradation fate and the end-of-life of the material, biodegradation tests in seawater, in the presence of microorganisms, and at various pH would be of relevance in the long term.

The *T*_g_ of PAM measured in this work (155 °C) agrees with reported values (135–170 °C) [50,51]. With increasing LA content, the polymer *T*_g_ shifted to lower values, which corresponds to literature observations, as the *T*_g_ of poly(LA) homopolymer was estimated to be −11 °C [43]. Due to the very high *T*_g_ of PAM, however, nearly all polymer products are extremely brittle at room temperature and were stored in the form of a powder after purification. The exception was the degraded AM/LA20, which exhibited a more malleable, plastic appearance. Its *T*_g_ fell from 81 °C to 3 °C after degradation due to its much lower molecular weight.

The original report describing controlled radical polymerization of LA used THF as solvent, but its solvent peak at 3.6 ppm in the ^1^H NMR spectrum overlapped with AM’s morpholine ring. Thus, MeCN was selected as an adequate solvent for PAM and AM/LA10. This highly polar solvent does not interfere with signals of interest during characterization using ^1^H NMR, and it was easy to remove during purification. With AM/LA20 however, phase separation began to occur at around 50% conversion in MeCN, as white precipitate started forming on the reactor wall. DMF was therefore selected as the polymerization solvent for AM/LA20. This observation served as an indicator of the limited water solubility of the copolymer made with a 20% LA feed.

LA is often referred to as a “universal antioxidant” for its quality of being both fat- and water-soluble [52]. Unfortunately, after ring-opening polymerization, LA repeat units within the chains seem to make the copolymer substantially more oily. Even at only 10 mol.% LA content, an aqueous solution of the copolymer manifested itself as a translucent dispersion at 700 ppm rather than a transparent solution. The fine suspension would settle at the bottom of the container after 24 h without stirring. Also at 700 ppm concentration, AM/LA20 formed clearly visible aggregates in water, which tended to adhere on the container wall even after 36 h of stirring. The higher LA composition resulted in its enhanced degradability, yet it became unsuitable for a KHI. This limitation was also observed in the literature with P(NIPAm-co-ester)s, obtained from ring-opening reactions of BMDO. Only copolymers with low BMDO content (less than 9 mol.%) were able to remain water-soluble [13].

**Figure 8:**
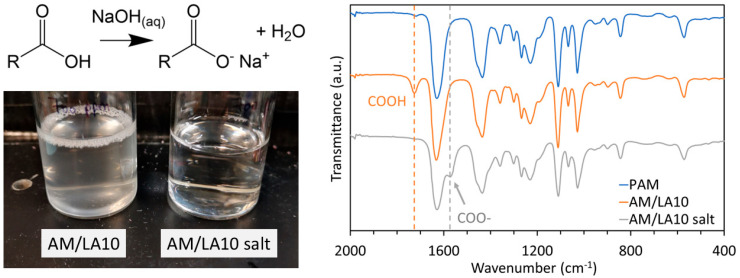
Comparison between AM/LA before and after the NaOH treatment. (**Left**) Appearance of 700 ppm solutions of AM/LA10 and its salt, and (**Right**) FTIR spectra of PAM, AM/LA10 and AM/LA10 salt.

Yet, aside from its affordability and accessibility, LA is also advantageous in terms of imparting versatility to the copolymers. Derivatives of carboxylic acid via nucleophilic substitution reactions, such as esters, amides, acid halides, and anhydrides, have been widely investigated [53]. In this manuscript, deprotonation of the carboxylic acid units using NaOH to form a sodium carboxylate salt was tested as a means to improve their water solubility. The comparison between the translucent AM/LA10 suspension before treatment and the clear solution after ionization is demonstrated in Figure 8 (Left). The clear solution appears significantly less soapy, as indicated by the persistent foam in AM/LA10, which was not occurring in its salt counterpart. Similarly, AM/LA20 has also become completely water-soluble. The FTIR spectra in Figure 8 (Right) shows the entire C=O stretch shifting from 1725 to 1572 cm^−1^ following the deprotonation. The DLS measurements are compiled in Table 2. The PAM homopolymer indicated a sub-10 nm hydrodynamic radius, which is typical for water-soluble polymers [54]. The z-average particle radius for AM/LA10 exceeded 10^2^ nm, confirming the presence of aggregates and that AM’s water solubility was compromised due to the addition of LA. In contrast, the particle size for AM/LA10 salt decreased back to ~ 10^1^ nm. Its value was larger than expected, as a hydrodynamic radius of ~30 nm falls within the range of polymer micelles [55]. However, its solution was completely transparent and colorless, displaying no haziness or blue shade due to scattered light, and thus the higher z-average size of the AM/LA carboxylate salt was likely due to the electrostatic interactions between the charged species [56]. Particle size was also measured from 0 to 70 °C, the highest operating temperature for the DTS0012 cuvettes used, and no thermoresponsive behavior was observed for these copolymers. A concentration of 1 wt.% was selected because the hydrate experiment concentration (700 ppm by weight) was too dilute to obtain accurate readings for the DLS.

**Figure 9:**
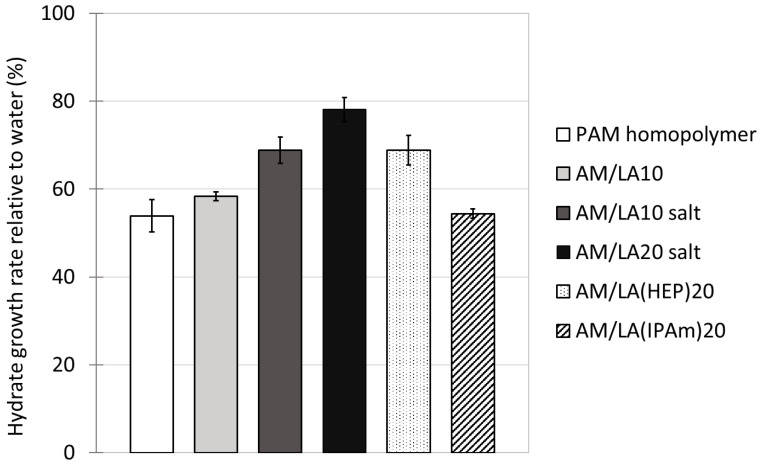
Methane consumption rate during hydrate growth in presence of various KHIs at a concentration of 700 ppm with respect to pure RO water control, at 4646 kPa and 2 °C, averaged over 5 runs, with 95% confidence interval error bars.

Using LA as a degradable comonomer therefore allows the production of highly water-soluble degradable copolymers. However, other challenges arise for the objective of applying these copolymers as hydrate inhibitors. The structure I methane hydrate growth rates with the addition of various KHIs at 700 ppm in RO water are tabulated in Figure 9. The effects of PAM and AM/LA10 reduced methane consumption in the crystallizer to 54% and 58% of that of the water control runs, respectively. The presence of LA within the statistical copolymer slightly reduced the inhibition performance at the cost of making the KHI degradable. Dividing the amide groups, which are crucial for hydrate inhibition, by inserting comonomers was unfavorable for the intended application. In their recent article, Kelland’s group attempted to use several polyesters as degradable polymeric KHIs, which also resulted in mild inhibitory effects [57]. It was suspected that the desire to introduce weaker linkages into the polymer backbone reduced the density of the crucial pendant-groups, typically amides. That hypothesis would also apply to this current study. The ring-opening polymerization of LA created more chain mobility, as characterized by DSC, which indicated lower *T*_g_s for the copolymers. As a result, this might create a looser barrier on the hydrate surface, or offer less steric hindrance during hydrate growth inhibition, as the gaps between side-chains are wider due to the insertion of disulfide bonds.

In addition, the inhibition performance of the completely water-soluble AM/LA salts are significantly weaker. Hydrate growth rates increased to 69% and 78% of those measured in water control runs for AM/LA10 salt and AM/LA20 salt, respectively. This notable decrease in KHI efficiency is likely to be correlated to their greater hydrophilicity. The only variation between AM/LA10 and its salt was the carboxylic acid and their corresponding carboxylate anion on the 10 mol.% LA repeat units. That alone raised gas consumption by approximately 10%, demonstrating the degree of sensitivity of hydrate growth to KHI molecular structure. Hydrophobicity of KHIs has long been an engaging topic of discussion [9,19,20,58]. Multiple recent works from Kelland’s group suggested that, between two very structurally similar repeat units, the one with a lower cloud point temperature tends to behave as the superior hydrate inhibitor [57,59,60,61]. The most well-known example is that of the 7-membered caprolactam ring in PVCap, which is more effective than the 5-membered pyrrolidone ring of PVP at delaying hydrate nucleation. It was theorized that PVCap’s bulkier side groups form a more rigid film than PVP when adsorbing on the hydrate surface, thus further reducing the addition of water molecules into the hydrate phase [62]. In contrast, PVA has demonstrated fairly weak inhibition effects compared to PVP, potentially due to its high hydrophilicity and shorter alkyl side groups, which yield less steric hindrance for water molecules to hydrogen bond onto the hydrate [9]. The LA comonomer might need some bulkier amide functionalities to become favorable for the desired application.

**Figure 10:**
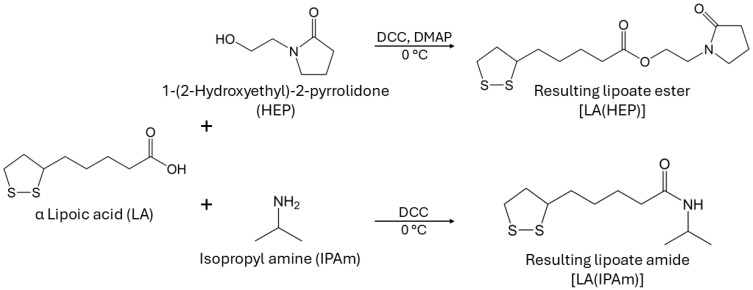
Coupling of lipoic acid with desirable functionalities for a KHI into LA(HEP) and LA(IPAm).

Since highly hydrophilic carboxylate salt end-groups are unsuitable for KHIs, another attempt was made to test different lipoic acid derivatives. Ester and amide couplings were carried out using 1-(2-Hydroxyethyl)-2-pyrrolidone (HEP) as the alcohol and isopropyl amine (IPAm) to produce LA(HEP) and LA(IPAm), respectively (Figure 10). The coupling reaction conditions and ^1^H NMR characterization of the new monomers and copolymers can be found in the Supporting Information. The TGA and DSC curves of the functionalized AM/LA copolymers, as well as GPC traces before and after reduction using TCEP, are also presented in the Supporting Information. Just as in the behavior of the AM/LA copolymer presented in Figure 6 and Figure 7, the functionalization did not impede LA’s compatibility with acrylamides during RAFT polymerization using the trithiocarbonate CTA, nor did it affect the copolymers’ ability for degradation. LA(HEP) and LA(IPAm) were able to be integrated at 20 mol.% within the copolymers with AM, also demonstrating lower onset temperatures and faster degradation in TGA, lower *T*_g_s in DSC, and lower *M*_n_ in GPC after disulfide reduction. Both the pyrrolidone group from HEP and the IPAm group are desirable pendant groups for a KHI. From the results in Figure 9, AM/LA(HEP)20 and AM/LA(IPAm)20 exhibited better performance than the AM/LA20 salt. Functionalizing the LA comonomer with amides was able to mitigate the issue of having “gaps” due to unfavorable side-groups within the polymer chain that do not participate in hydrate inhibition.

Although being an improvement over the carboxylate salt, AM/LA(HEP)20 was still not particularly beneficial as a KHI, being poorer than the PAM homopolymer. The issue could lie within its overly long side-chain. Literature suggests that these “dangling” functional groups at the end of a long side-chain might result in a more positive Gibbs free energy upon interaction with the hydrate surface [59]. Thus, adsorption and formation of a sturdy barrier between the hydrate and bulk aqueous phases might not be favorable. Its increased mobility is reflected in the liquid state of the LA(HEP) monomer at room temperature as opposed to the yellow powder for LA and LA(IPAm). The DSC curves in the Supporting Information also indicate a much lower *T*_g_ for AM/LA(HEP)20 at 39 °C compared to that of AM/LA20 and AM/LA(IPAm)20, which were both above 80 °C. It is hypothesized that the high chain mobility and flexibility, associated with the measurably lower *T*_g_, would lead to a less rigid and persistent film on the crystal surface, therefore reducing the inhibition efficacy of the HEP-functional lipoate copolymer.

In contrast, the IPAm-functionalized LA copolymer obtained more promising results, reaching a 54% methane intake rate relative to the water reference. AM/LA(IPAm)20 had nearly re-established the performance of the PAM homopolymer by functionalizing the degradable comonomer with a short and effective amide. The addition of IPAm seems to have counteracted the aforementioned undesirable factors for a hydrate inhibitor, such as decreased amide density and increased chain mobility of the copolymers. It is proposed that these rigid IPAm groups filled in the “gaps” of inhibitory amide moieties lost due to the dilution of acrylamide repeat-units, and that they were able to recover the necessary steric hindrance and surface adsorption during crystal inhibition. In the future, attempts to copolymerize functionalized LA with vinyl amides such as NVP and to verify their compatibility are of interest, notably using RAFT agents such as dithiocarbamates and xanthates, appropriate for less-activated monomers.

The modification into LA(IPAm) has taken a step into a promising direction for lipoate-based degradable copolymers for KHIs. Multiple challenges have been overcome; however, they are not without limitations. For instance, at a concentration of 1 wt.% in RO water, both functionalized LA copolymers exhibited the form of a fine milky suspension, which would settle at the bottom of the container after prolonged times without stirring. Although both are more water-soluble than AM/LA, as the latter could only form large aggregates in water once LA composition reaches 20 mol.%, the functionalized non-ionic LA copolymers were not able to attain the degree of water-affinity that the PAM homopolymer or the AM/LA salt possess. More challenges would arise if the functionalized LA were to be introduced into KHIs with lower cloud-point temperatures, such as PVCap and PNIPAm. A potential solution would be to tune the copolymers’ water-solubility by combining both the carboxylate and amide functional groups. Nevertheless, even in the case where the LA comonomer cannot be integrated at high concentration in order to preserve the desirable properties of the original homopolymer, presence of weaker S-C bonds at less than 5 mol.% LA content is still more favorable compared to the stable C-C bonds in terms of degradation [42].

Finally, in terms of copolymers as KHIs, the different repeat units within a copolymer are ideally able to be synergetic during hydrate inhibition, and to perform even better than the homopolymers, likely due to a greater degree of disruption of water’s hydrogen-bonding network [63]. Literature reports have developed various copolymers of PVCap which possess enhanced properties compared to the PVCap homopolymer [64,65]. Unfortunately, in this case, the relatively long alkane chains of the LA backbone and pendant-group limited its potential as a synergetic comonomer with the statistical microstructure. Another way to improve inhibition performance of lipoate-based KHIs would be to investigate the block copolymer microstructure. Researchers have tested multiple amphiphilic block copolymers as KHIs, and they have demonstrated significant improvement in terms of delaying hydrate nucleation or reducing gas consumption during the growth phase compared to PVP, PVCap, or poly(meth)acrylamides homopolymers [9,11,40,66,67,68]. Notably, a partially degradable ε-caprolactone/PVCap copolymer was able to enhance the performance of PVCap as KHI. In another very recent study, a block copolymer of an ethyl lipoate ester derivative with styrene was made possible by chain-extending a thiol-terminated PS mCTA [45]. The oily nature of polymerized LA can be exploited, and the synthesis of an LA/acrylamide (or vinyl amide) amphiphilic block copolymer may lead to promising future studies.

## 4. Conclusions

LA, being an affordable and non-toxic compound, can introduce weak S-S and S-C linkages into the otherwise chemically stable C-C vinyl polymer backbone. Statistical copolymers of AM with LA were synthesized via RAFT polymerization using trithiocarbonate CTA, with the objective of creating water-soluble polymers with the ability to both inhibit hydrate formation and to degrade under mild conditions. Compared to the PAM homopolymer, the AM/LA copolymers, up to 20 mol.% LA content, demonstrated more rapid weight reduction during TGA tests, lower *T*_g_s in DSC experiments, and a shift towards lower *M*_n_ measured by GPC upon TCEP disulfide reduction.

While the PAM homopolymer and LA monomers are water-soluble, polymers containing LA after ring-opening polymerization became substantially more oily. As the number of degradation sites increased within the polymer chain, water solubility drastically decreased, creating aggregates in aqueous solutions with only 20 mol.% LA content within the copolymer. Although LA can be deprotonated to form lipoate salts, which greatly enhanced its hydrophilicity, the ionization impeded the copolymer’s ability to inhibit hydrate growth. Providentially, another advantage of LA is its high versatility. An ester and an amide derivatives of LA were synthesized using HEP and IPAm, respectively. They allowed an improvement in water-solubility and in hydrate inhibition performance compared to the original LA copolymers, without hindering their ability to degrade, nor their compatibility with acrylamides during RAFT copolymerization.

The ring-opening polymerization of the disulfide-containing degradable comonomer led to decreased hydrate inhibition, likely due to increased chain mobility and a decrease in amide density within the polymer chain, which potentially impeded the copolymer’s ability to adhere onto the hydrate surface and efficiently reduce the mass transfer of gas and water molecules into the hydrate phase. By coupling LA with IPAm, the resulting degradable copolymer was able to re-establish its KHI performance compared to the acrylamide homopolymer.

The amide-derivative of LA acted as the most promising building block for degradable KHIs, likely because the short and rigid IPAm moieties were able to compensate for the steric hindrance and surface adhesion lost due to incorporating LA repeat-units within the amide-based polymeric inhibitor. Future works will include verifying the compatibility of this degradable monomer during RAFT copolymerization with vinyl amides, in contrast to the acrylamide tested in this study.

## Figures and Tables

**Table 2:** DLS results for z-average particle size of PAM, AM/LA10 and AM/LA10 salt at 25 °C and concentration of 1 wt.% in RO water, taking the mean of 3 measurements, with 12 runs per measurement.

Polymer ID	Mean Z-Average (Radius, nm)	Standard Deviation (Radius, nm)
PAM	9.1	0.2
AM/LA10	585.6	33.8
AM/LA10 salt *	28.4	0.1

* Accuracy of DLS readings might be compromised by electrostatic interactions due to the charged carboxylate.

## Data Availability

The data presented in this study are openly available in Borealis–McGill University Dataverse at https://doi.org/10.5683/SP3/YFANDE.

## Supplementary Materials

The following supporting information can be downloaded at https://www.mdpi.com/article/10.3390/polym17233125/s1: Figure S1: ^1^H NMR spectrum of lipoic acid, 1-(2-hydroxyethyl)-2-pyrrolidone, and the resulting lipoate ester LA(HEP) following the coupling, in CDCl_3_ and 500 MHz; Figure S2: ^1^H NMR spectrum of AM/LA(HEP)20 statistical copolymer, in CDCl_3_ and 500 MHz; Figure S3: ^1^H NMR spectrum of lipoic acid, isopropyl amine, and the resulting lipoate amide LA(IPAm) following the coupling, in CDCl_3_ and 500 MHz; Figure S4: ^1^H NMR spectrum of AM/LA(IPAm)20 statistical copolymer, in CDCl_3_ and 500 MHz; Figure S5: GPC traces of AM/LA(HEP)20 and AM/LA(IPAm)20 before and after TCEP reduction, samples eluted in THF at 40 °C, *M*_n_ and *Ð* calculated using a PMMA calibration curve; Figure S6: Left: TGA heating ramps showing lower onset temperatures for AM/LA(HEP)20 and AM/LA(IPAm)20 compared to PAM homopolymer, performed under N2 and at 15 °C/min. Right: DSC 2nd heating curves showing lower *T*_g_s for the functionalized AM/LA copolymers, as well as even lower *T*_g_s for the degraded copolymer, heat–cool–heat experiments performed under N2 and at 15 °C/min.
