# Supplementary material for: Evaluation of Acrylamide/α-Lipoic Acid Statistical Copolymers as Degradable Water-Soluble Kinetic Gas Hydrate Inhibitors
Source: Polymers (Basel). 2025 Nov 25;17(23):3125. doi: 10.3390/polym17233125 (PMC12694544; doi:10.3390/polym17233125)
Supplement: Supplementary file 1 [file polymers-17-03125-s001.zip › polymers-3962092-supplementary.pdf]

Supporting information for:

Evaluation of Acrylamide/α-Lipoic Acid Statistical Copolymers as Degradable Water-Soluble Kinetic Gas Hydrate Inhibitors

*Chong Yang Du, Milan Marić, Phillip Servio**

Department of Chemical Engineering, McGill University, Montreal, Quebec H3A 0C5, Canada

*Email: phillip.servio@mcgill.ca

Table of Contents

Figure S1. ^{1}H NMR spectrum of lipoic acid, 1-(2-hydroxyethyl)-2-pyrrolidone, and the resulting lipoate ester LA(HEP) following the coupling, in $CDCl_3$ and 500 MHz. 3

Figure S2. ^{1}H NMR spectrum of AM/LA(HEP)20 statistical copolymer, in $CDCl_3$ and 500 MHz. 4

Figure S3. ^{1}H NMR spectrum of lipoic acid, isopropyl amine, and the resulting lipoate amide LA(IPAm) following the coupling, in $CDCl_3$ and 500 MHz... 5

Figure S4. ^{1}H NMR spectrum of AM/LA(IPAm)20 statistical copolymer, in $CDCl_3$ and 500 MHz. 6

Figure S5. GPC traces of AM/LA(HEP)20 and AM/LA(IPAm)20 before and after TCEP reduction, samples eluted in THF at 40 °C, *M*n and *Đ* calculated using a PMMA calibration curve. 7

Figure S6. Left: TGA heating ramps showing lower onset temperatures for AM/LA(HEP)20 and AM/LA(IPAm)20 compared to PAM homopolymer, performed under N_2 and at 15 °C/min. Right: DSC 2nd heating curves showing lower T_gs for the functionalized AM/LA copolymers, as well as even lower T_gs for the degraded copolymer, heat-cool-heat experiments performed under N_2 and at 15 °C/min. 7

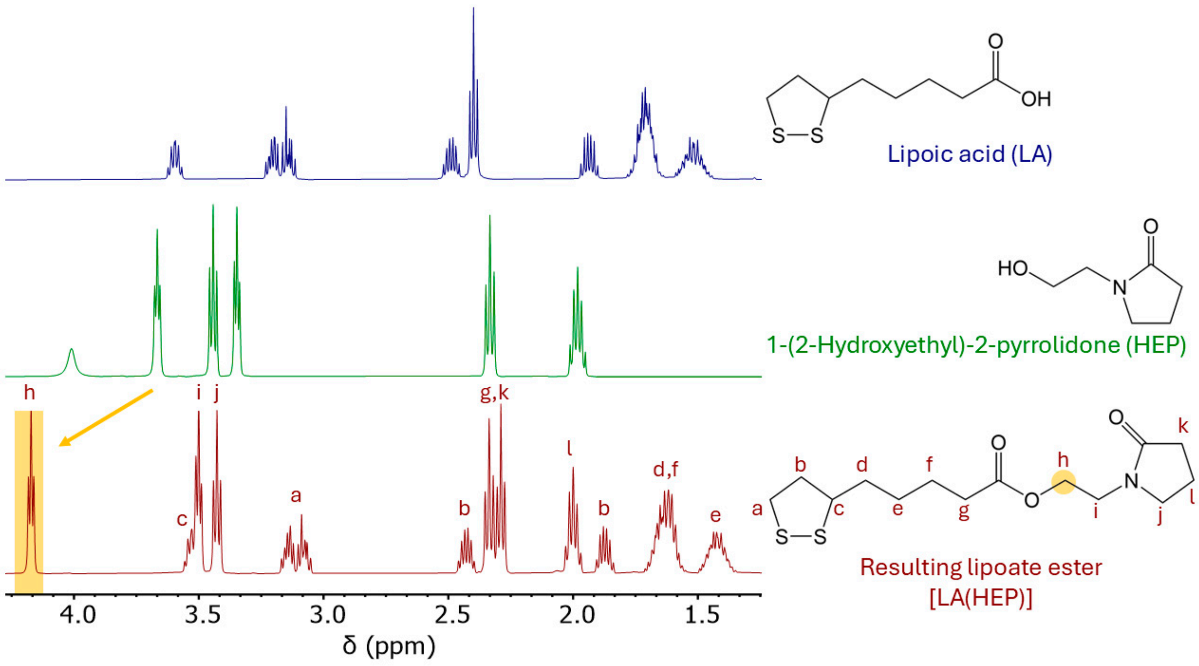

Figure S1. ^{1}H NMR spectrum of lipoic acid, 1-(2-hydroxyethyl)-2-pyrrolidone, and the resulting lipoate ester LA(HEP) following the coupling, in $CDCl_3$ and 500 MHz.

Ester coupling and purification methodology

In a round-bottom reactor submerged in an ice bath, 15 g of LA (0.0727 mol, 1 mol equivalent) and 26.76 g of HEP (0.207 mol, 2.85 mol equivalent) were dissolved in 60 mL of THF. In a different beaker, 15 g of *N*,*N′*-dicyclohexylcarbodiimide (DCC, 1 mol equivalent) was dissolved in 60 mL of THF. The DCC solution was added to the LA+HEP solution and left for stirring for 10 minutes until the mixture became hazy. Then, 0.888 g of 4-dimethylaminopyridine (DMAP, 0.1 mol equivalent) was dissolved in 30 mL of THF. This third solution was added dropwise into the round-bottom reactor over 40 minutes. The reaction was left to complete at room temperature overnight. The THF was evaporated using a rotary evaporator. The reaction product was purified by eluting through a silica gel column using ethyl acetate (EtAc) as eluent. The resulting lipoate ester LA(HEP) monomer presents itself in the form of a yellow oil.

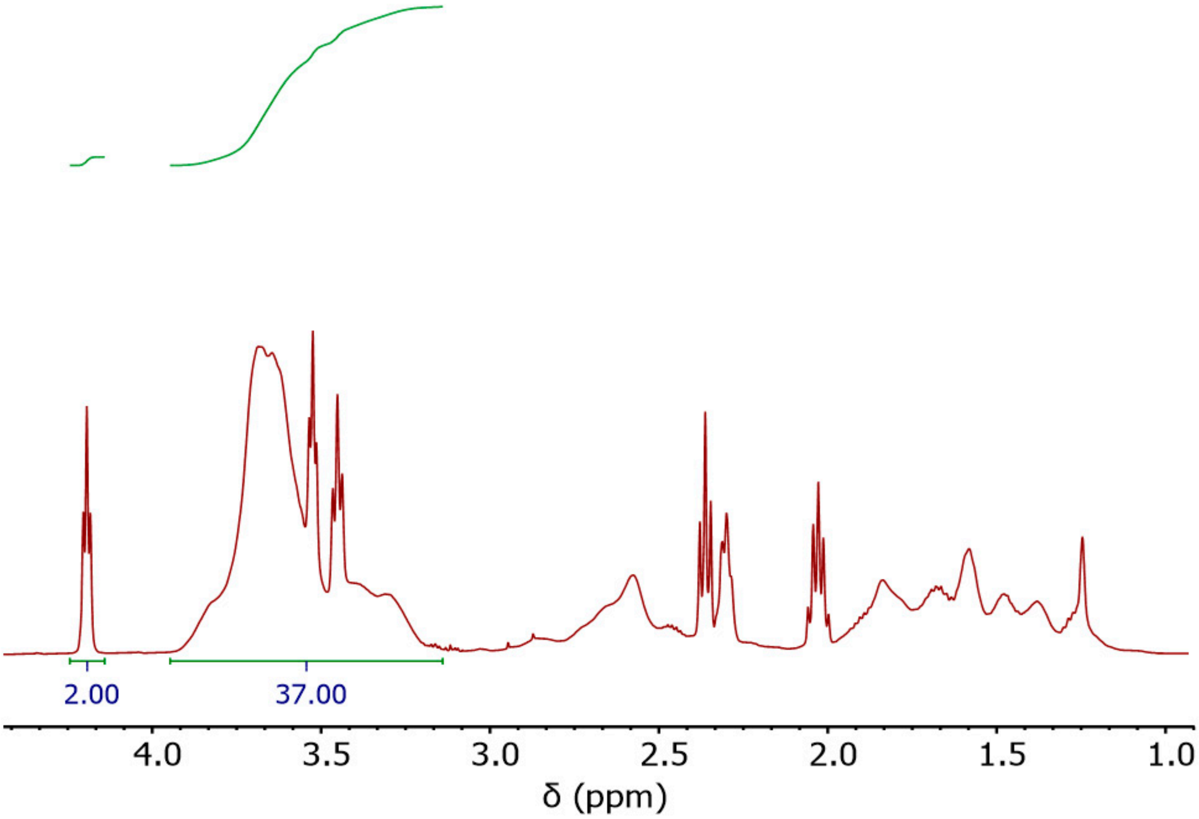

Figure S2. ^{1}H NMR spectrum of AM/LA(HEP)20 statistical copolymer, in $CDCl_3$ and 500 MHz.

δ = 4.20 (2H poly(LA(HEP)), ester O-CH_2-CH_2-N), 3.95-3.14 (broad, 8H poly(AM), morpholine ring), 3.53 (2H poly(LA(HEP)), O-CH_2-CH_2-N), 3.45 (2H poly(LA(HEP), N-CH_2 in the pyrrolidone ring).

Calculation of AM/LA(HEP)20 copolymer molar composition:

$$F_{\text{LA(HEP)}} = \frac{\frac{2.00}{2}}{\frac{2.00}{2} + \frac{37.00 - 2.00 - 2.00}{8}} \times 100\% = 19.5\%$$

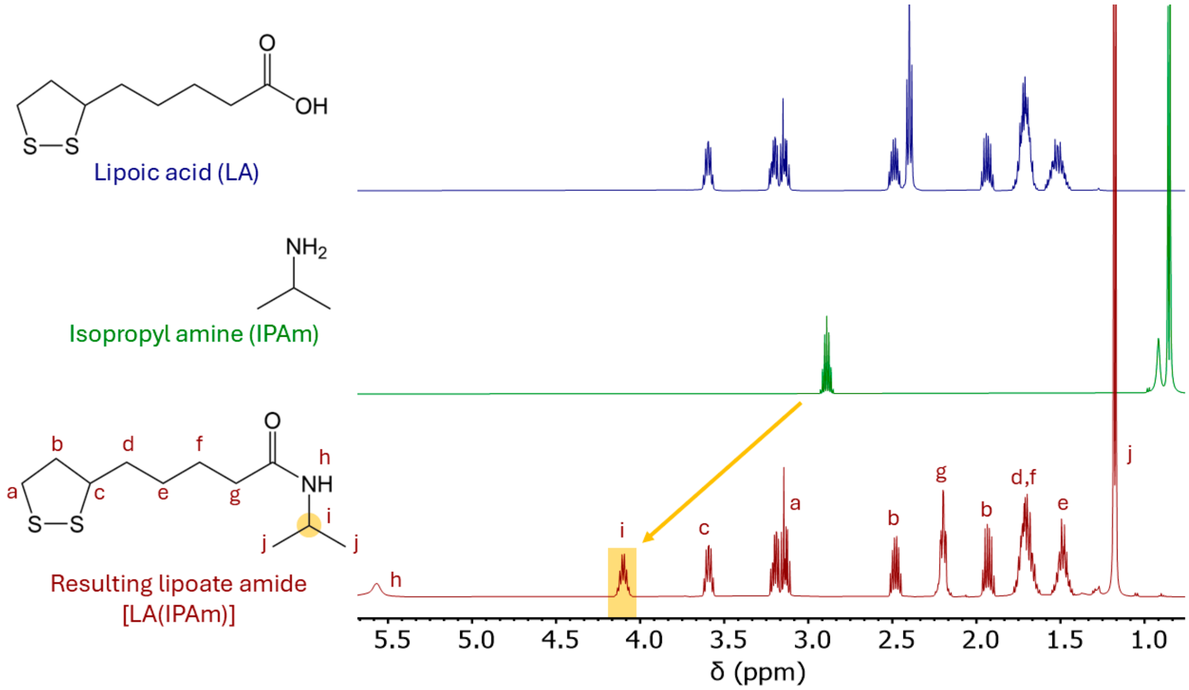

Figure S3. ^{1}H NMR spectrum of lipoic acid, isopropyl amine, and the resulting lipoate amide LA(IPAm) following the coupling, in $CDCl_3$ and 500 MHz.

Amide coupling and purification methodology

In a round-bottom reactor submerged in an ice bath, 10 g of LA (1 mol equivalent) was dissolved in 60 mL of DCM. In a different beaker, 12 g of DCC (1.2 mol equivalent) was dissolved in 60 mL of DCM. The DCC solution was added to the LA solution and left for stirring for 10 minutes until the mixture became hazy. Then, 16.7 mL of IPAm (4 mol equivalent) was diluted in 30 mL of DCM. This third solution was added dropwise into the round-bottom reactor over 30 minutes. The reaction was left to complete at room temperature overnight. The DCM mixture was washed using 100 mL of brine, 1M $KHSO_4$ solution, brine, 5 wt.% $NAHCO_3$, and brine. After solvent evaporation, a pale-yellow solid was left in the reactor. It was dissolved in a minimal amount of 50/50 vol.% DCM/EtAc solvent mixture and eluted through a silica gel column using

EtAc. The product was recovered by recrystallization using hexanes and dried under vacuum. The resulting lipoate amide LA(IPAm) monomer presents itself in the form of a yellow powder.

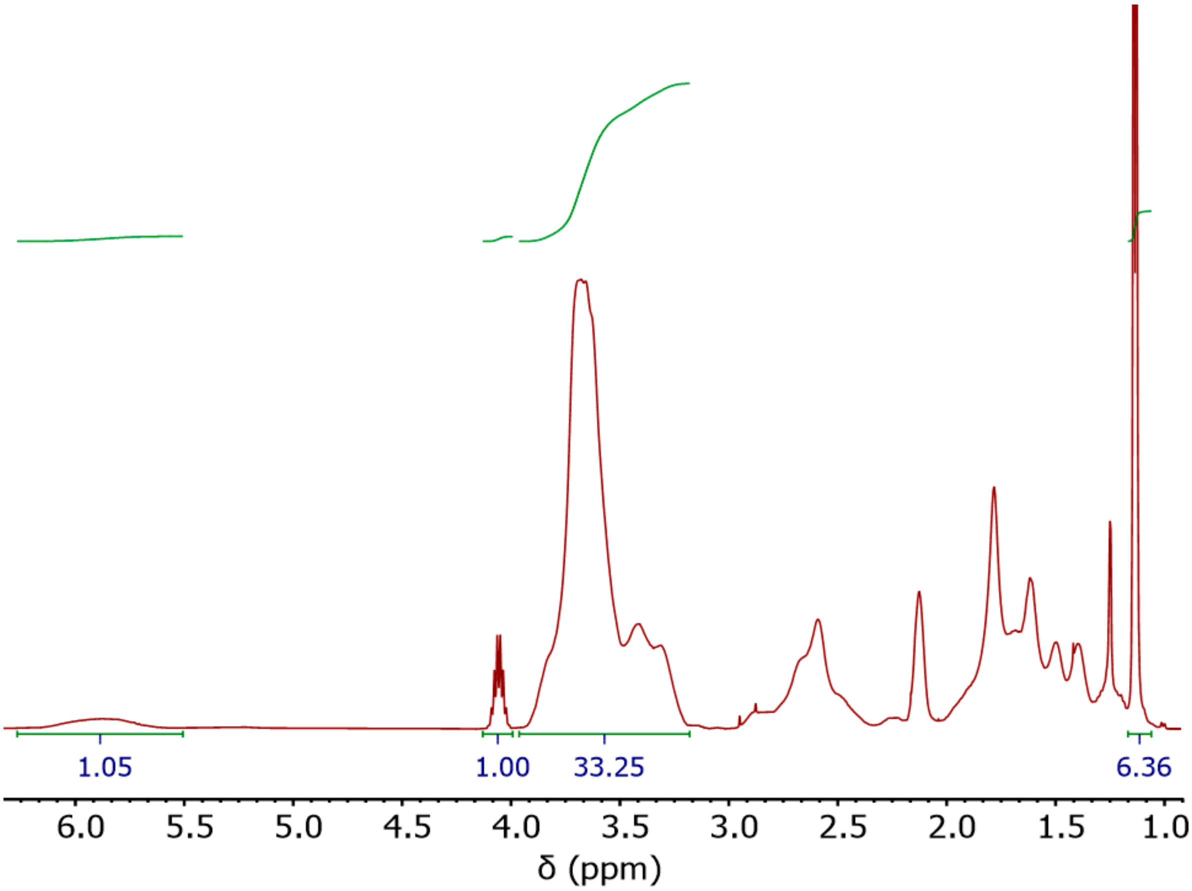

Figure S4. ^{1}H NMR spectrum of AM/LA(IPAm)20 statistical copolymer, in $CDCl_3$ and 500 MHz. δ = 6.27-5.51 (broad, 1H poly(LA(IPAm)), N*H*-CH-$(CH_3)_2$), 4.06 (1H poly(LA(IPAm)), NH-C*H*-$(CH_3)_2$), 3.96-3.17 (broad, 8H poly(AM), morpholine ring), 1.15 (6H poly(LA(IPAm)), NH-CH-$(CH_3)_2$).

Calculation of AM/LA(IPAm)20 copolymer molar composition:

$$F_{\mathrm{LA(IPAm)}} = \frac{1.00}{1.00 + \frac{33.25}{8}} \times 100\% = 19.4\%$$

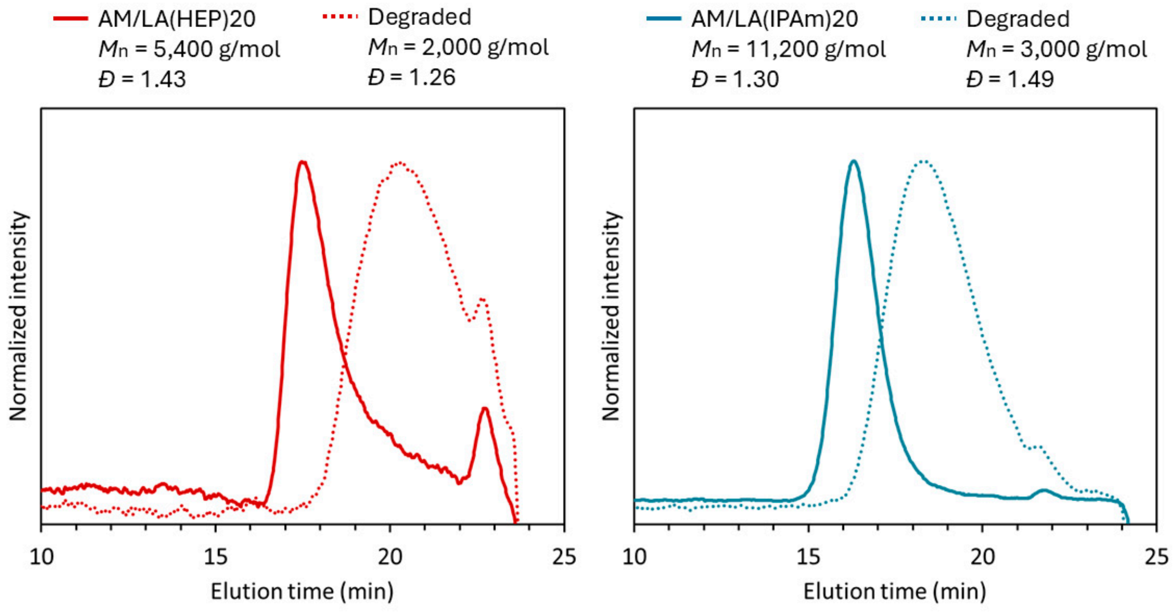

Figure S5. GPC traces of AM/LA(HEP)20 and AM/LA(IPAm)20 before and after TCEP reduction, samples eluted in THF at 40 °C, M_n and $Đ$ calculated against a PMMA calibration curve.

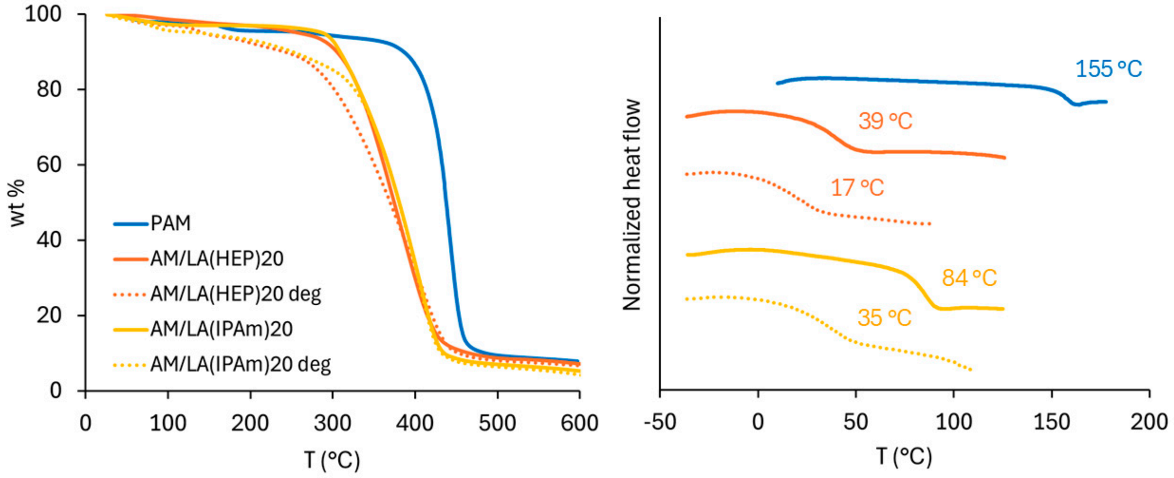

Figure S6. Left: TGA heating ramps showing lower onset temperatures for AM/LA(HEP)20 and AM/LA(IPAm)20 compared to PAM homopolymer, performed under N_2 and at 15 °C/min. Right: DSC 2nd heating curves showing lower T_gs for the functionalized AM/LA copolymers, as well as even lower T_gs for the degraded copolymers, heat–cool–heat experiments performed under N_2 and at 15 °C/min.
